# Pacing strategy patterns and performance outcomes in marathon running: A large-scale analysis of split time data

**DOI:** 10.1371/journal.pone.0352808

**Published:** 2026-07-17

**Authors:** Qizheng Dong, Zaidong Li

**Affiliations:** 1 School of Physical Education, Zhengzhou University of Science and Technology, Zhengzhou, China; 2 School of International Education, Henan University of Engineering, Zhengzhou, China; University of Mississippi, UNITED STATES OF AMERICA

## Abstract

Although pacing strategy is widely recognized as critical for marathon performance, the actual distribution of pacing patterns among recreational runners and their associations with finish time across demographic groups have not been systematically characterized in large samples. This study analyzed split time data (i.e., cumulative elapsed times recorded at intermediate checkpoints including 5K, 10K, 15K, 20K, half-marathon, 25K, 30K, 35K, and 40K) from 78,912 finishers of the Boston Marathon (2015–2017), characterizing pacing profiles using the ratio of first-half to second-half completion times and segment-by-segment pace variations across these nine timing points. Cluster analysis was employed to identify distinct pacing patterns, and the relationships between pacing strategy and finish time were examined across age groups, sex, and performance levels using analysis of variance and multiple regression analysis. Four distinct pacing patterns were identified: even-pacing (maintaining consistent pace throughout; 47.9%), mild positive-splitting (controlled late-race slowing; 31.9%), strong positive-splitting (pronounced late-race slowing; 12.2%), and variable-pacing (highly inconsistent pace fluctuations; 8.0%). Even-pacing was consistently associated with the fastest finish times across age and performance strata (*p* < 0.001), with mean finish times markedly lower than those observed for strong positive-splitting (217 vs. 276 minutes). Variable-pacing was associated with the highest incidence of “hitting the wall” (pace decline >20% in the final segment; 85.4%). High-performance runners (<3:00 finish time) demonstrated significantly higher rates of even-pacing (82.0%) compared to casual participants (19.6%). Age and sex significantly influenced pacing strategy selection (*p* < 0.001). These findings may help inform marathon pacing guidance by indicating that age, sex, and performance level should be considered when interpreting pacing patterns and developing individualized race plans.

## Introduction

Pacing strategy, defined as the distribution of running speed throughout a race, is widely recognized as a critical determinant of endurance performance [1,2]. In marathon running, the selection and execution of an appropriate pacing strategy can substantially influence both finish time and the overall race experience [3,4]. The marathon distance of 42.195 kilometers presents unique physiological and psychological challenges that make pacing particularly important, as runners must carefully manage finite energy reserves over an extended duration typically lasting between two and six hours [5].

One of the most commonly discussed phenomena in marathon running is “hitting the wall,” a dramatic decline in running pace typically occurring in the final third of the race [6,7]. This phenomenon is primarily attributed to glycogen depletion in skeletal muscles and the liver, forcing the body to rely more heavily on fat oxidation for energy production, which cannot sustain the same running velocities as carbohydrate metabolism [8,9]. Large-scale observational studies of race results suggest that a substantial proportion of recreational marathon runners experience some degree of pace decline in the latter stages of the race, with severe cases resulting in significant performance impairment and increased injury risk [10,11].

The importance of pacing strategy extends beyond elite competition to encompass the vast majority of recreational marathon participants [12,13]. With global marathon participation reaching unprecedented levels—exceeding 7 million annual finishers worldwide—the development of evidence-based pacing guidelines has become increasingly relevant for runners of all ability levels [12,13]. Optimal pacing strategies can help runners achieve their performance potential while minimizing the physiological and psychological distress associated with premature fatigue [14].

Research in endurance sports has identified several distinct pacing strategy categories, each characterized by a specific pattern of speed distribution throughout the race [1]. **Even-pacing** involves maintaining a relatively constant velocity throughout the race, with minimal variation between split times. This strategy is theoretically optimal from a bioenergetic perspective, as it minimizes the accumulation of metabolic byproducts and optimizes glycogen utilization [15]. Elite marathon runners and world record performances have typically been characterized by remarkably even pacing, with first-half and second-half times differing by less than 1–2% (i.e., a half-marathon ratio *R*_half_ between 0.98 and 1.02) [1,16]. **Positive-splitting** refers to a pacing pattern in which the first half of the race is completed faster than the second half. This strategy often results from starting too aggressively relative to one’s fitness level, leading to premature glycogen depletion and progressive fatigue accumulation [17]. Positive-splitting is commonly observed among recreational runners and is associated with slower overall finish times and higher rates of “hitting the wall” [7,18]. **Negative-splitting** describes a pacing strategy in which the second half of the race is completed faster than the first half. While theoretically attractive as a conservative approach that preserves energy for the final stages, negative-splitting is relatively rare in marathon running and requires precise fitness assessment and exceptional pacing discipline [3]. Some research suggests that mild negative-splitting may be associated with optimal performance in certain conditions [19]. **Variable-pacing** encompasses strategies characterized by substantial fluctuations in running speed throughout the race, operationally defined in our analyses as pacing variability with CV_pace_ > 15%. This pattern may result from course topography, environmental conditions, tactical racing decisions, or inconsistent effort regulation [1,20]. Variable-pacing is generally considered suboptimal for time-trial events like marathon running, where steady-state effort is typically most efficient [21].

The physiological mechanisms underlying these pacing strategies involve complex interactions between central neural regulation, peripheral muscle fatigue, and metabolic substrate availability [14,22]. The central governor model proposes that the brain continuously integrates afferent feedback from multiple physiological systems to regulate exercise intensity and prevent catastrophic physiological failure [23].

The scientific study of pacing strategies in endurance sports has evolved substantially over the past three decades, yet fundamental questions regarding optimal pacing for marathon running remain incompletely resolved [1,4]. Early theoretical work by Foster and colleagues established the bioenergetic rationale for even-pacing strategies, demonstrating that constant power output minimizes the overall metabolic cost of completing a fixed distance [15]. This principle, derived from mathematical models of energy expenditure, suggested that any deviation from a constant pace would result in suboptimal performance due to the nonlinear relationship between velocity and energy cost [15]. However, the translation of these theoretical principles to practical marathon racing has proven more complex than initially anticipated. Tucker and Noakes proposed the central governor model, which reconceptualized pacing as a regulated behavior controlled by the brain to prevent catastrophic physiological failure [14,23]. This framework suggested that pacing decisions are made anticipatorily based on prior experience, expected race duration, and real-time feedback from peripheral systems [14]. While this model provided valuable insights into the neural regulation of exercise intensity, it offered limited practical guidance for how runners should distribute their effort across different race segments [1,4]. The teleoanticipation model advanced by Ulmer further emphasized the role of feedforward mechanisms in pacing regulation, proposing that athletes continuously adjust exercise intensity based on projected endpoints and accumulated fatigue [1,24]. Despite these theoretical advances, a significant gap persists between laboratory-based pacing models and the empirical realities of marathon racing, where course topography, environmental conditions, competitive dynamics, and psychological factors all influence pacing decisions in ways that idealized models cannot fully capture [20,25].

Empirical research on marathon pacing has produced valuable insights but has been constrained by methodological limitations that restrict the generalizability of findings. The seminal analysis by Abbiss and Laursen comprehensively reviewed pacing strategies across endurance sports and established the conceptual framework of even, positive, negative, and variable pacing that continues to guide research today [1]. Their work synthesized evidence demonstrating that elite marathon performances typically exhibit even-pacing characteristics, with minimal variation between the first and second halves of the race. Subsequent studies have largely confirmed these observations while revealing important nuances. Santos-Concejero and colleagues examined pacing profiles in elite marathon runners and found that the most successful performances were characterized by negative-splitting or even-pacing strategies, with positive-splitting associated with progressively slower finishing positions [16]. Hanley’s analysis of World Half Marathon Championship data demonstrated similar patterns, with pack running and drafting introducing additional tactical considerations that can influence pacing decisions [3]. These elite-focused studies have contributed substantially to understanding optimal pacing in high-performance contexts.

Nevertheless, the overwhelming concentration of research on elite athletes represents a critical limitation of the existing literature. Recreational runners, who constitute the vast majority of marathon participants, face qualitatively different physiological and psychological challenges than their elite counterparts [12,13]. The margin for pacing error is substantially wider for slower runners, who spend longer on the course and are more vulnerable to glycogen depletion, dehydration, and thermal stress [12,13]. Studies examining recreational populations remain relatively scarce, and those that exist have typically employed small samples or limited pacing metrics that may obscure meaningful patterns of pace distribution [12,13,25–27]. Moreover, the methodological approaches used to classify pacing strategies have often relied on predetermined categorical definitions rather than data-driven identification of naturally occurring patterns. Most studies have operationalized pacing using the ratio of first-half to second-half time, while ignoring potentially important information contained in segment-by-segment split data [28] and the consistency of pacing within each segment [29]. This reductionist approach may miss nuanced pacing behaviors that emerge only when analyzing complete temporal profiles of pace distribution.

A growing body of research has examined how individual characteristics influence pacing strategy adoption and the relationship between pacing and performance outcomes. Sex differences in marathon pacing have received considerable attention, with Deaner and colleagues demonstrating that men are more likely than women to exhibit positive-splitting and experience significant late-race pace decline [18]. This finding has been attributed to various factors, including differences in risk-taking behavior, metabolic substrate utilization patterns, and socialized competitive tendencies, though the precise mechanisms remain debated. Age-related changes in pacing behavior have also been documented, with older runners generally demonstrating more conservative pacing strategies and lower rates of severe late-race pace decline [12,26]. These observations align with theoretical predictions that running experience promotes better calibration of pacing decisions, though the confounding effects of physiological aging and self-selection into marathon participation complicate interpretation [25,27]. The interaction between age and other individual factors such as training history and competitive orientation remains largely unexplored.

The dramatic late-race pace decline colloquially termed “hitting the wall” has long fascinated both researchers and the running community, yet scientific understanding of this phenomenon remains incomplete. Buman and colleagues estimated that approximately 40% of marathon runners experience some form of significant late-race deterioration, though definitions and incidence rates vary considerably across studies [6]. Rapoport’s metabolic modeling suggested that hitting the wall occurs when glycogen stores become critically depleted, forcing a metabolic shift toward fat oxidation that cannot sustain high running velocities [8]. Smyth and colleagues employed large-scale data analysis to examine pace decline patterns in marathon running and confirmed that excessive early pacing was strongly predictive of severe late-race deceleration [10]. Their work represented an important methodological advance by leveraging comprehensive timing data from major marathon events, though the analysis focused primarily on aggregate patterns rather than individual-level risk factors or the identification of distinct pacing subtypes.

Despite the recognized importance of pacing strategy in marathon performance, several significant limitations characterize the existing research literature. First, several previous studies have been constrained by relatively small sample sizes, typically examining hundreds to a few thousand participants from single race events [13,25,30]. These limited samples may not adequately represent the diversity of pacing behaviors across different demographic groups, performance levels, and race conditions. Second, the classification of pacing strategies in previous research has often relied on subjective or arbitrary criteria, such as defining positive-splitting based on fixed percentage thresholds without empirical validation [19]. Data-driven approaches using cluster analysis or other pattern recognition methods have been underutilized, potentially missing important nuances in pacing behavior that do not conform to predefined categories [2,19]. Third, most pacing research has focused on elite or sub-elite runners, with less attention devoted to recreational participants who constitute the majority of marathon fields [7,18]. Fourth, the influence of individual characteristics such as age, sex, and experience on optimal pacing strategy selection remains incompletely understood [12,31]. Fifth, longitudinal data examining the “hitting the wall” phenomenon through detailed segment-by-segment analysis have been limited [6,10].

The proliferation of electronic timing systems and public availability of race results has created unprecedented opportunities for large-scale analysis of athletic performance, yet this potential remains substantially underutilized in marathon pacing research. While studies analyzing thousands of participants have become more common, the application of contemporary pattern recognition methods to pacing data has been limited [12,26]. Cluster analysis and related unsupervised learning techniques offer particular promise for identifying naturally occurring pacing patterns without imposing predetermined categorical structures. These methods have been successfully applied in other domains of sports science but have seen limited adoption in marathon pacing research [2,19]. The continued reliance on simplistic half-marathon ratio metrics, when segment-by-segment timing data are readily available, represents a methodological limitation that this study seeks to address.

To address these limitations, this study analyzed detailed split time data—comprising cumulative elapsed times recorded at nine intermediate checkpoints (5K, 10K, 15K, 20K, half-marathon, 25K, 30K, 35K, and 40K)—from 78,912 finishers of the Boston Marathon across three consecutive years (2015–2017). The Boston Marathon represents an ideal context for pacing research due to its status as a qualifying race with established performance standards, its challenging point-to-point course profile, and the availability of comprehensive timing data at nine intermediate checkpoints within the publicly available 2015–2017 curated dataset used here. The specific objectives of this study were: (1) to identify distinct pacing strategy patterns using data-driven cluster analysis of segment-by-segment split times; (2) to examine the associations between identified pacing patterns and marathon finish time across different performance levels; (3) to investigate how demographic factors (age, sex) influence pacing strategy adoption and optimal pacing-performance relationships; and (4) to characterize the “hitting the wall” phenomenon through analysis of late-race pace decline patterns.

Based on previous research and physiological principles, we hypothesized that: (H1) distinct pacing patterns would emerge from cluster analysis, corresponding broadly to even-pacing, progressively stronger forms of positive-splitting, and variable-pacing; (H2) even-pacing would be associated with significantly faster finish times compared to other pacing strategies across all performance levels; (H3) strong positive-splitting would be more prevalent among recreational runners compared to high-performance and sub-elite participants; and (H4) the incidence of “hitting the wall” would be significantly higher among runners adopting positive-splitting-derived patterns compared to even-pacing strategies.

## Materials and methods

### Study design

This study employed a retrospective cross-sectional design to analyze pacing strategies and performance outcomes in marathon running. The investigation utilized publicly available race results from the Boston Marathon, one of the world’s most prestigious and longest-running annual marathon events. This observational approach allowed for the examination of naturalistic pacing behaviors across a large and diverse sample of marathon finishers without the constraints of experimental manipulation.

The Boston Marathon was selected as the data source for several methodological advantages. First, as a qualifying race, participants must meet age- and sex-specific time standards in a previous marathon, ensuring a sample with demonstrated endurance running competence. Second, the race employs professional chip timing with multiple intermediate checkpoints, providing high-quality split time data essential for detailed pacing analysis. Third, the consistent point-to-point course and annual timing in mid-April reduce variability attributable to course design and seasonal factors. Given the public nature of the race results and the absence of individually identifiable health information beyond age and sex, formal ethical approval was not required for this secondary analysis of publicly available data.

### Participants

The study sample comprised all finishers of the Boston Marathon across three consecutive years from 2015 to 2017, representing a total of 79,638 completed race records. For each finisher, the dataset provided complete split time data at nine intermediate timing points, enabling detailed segment-by-segment pacing analysis rather than reliance on finish time alone or a small number of checkpoints. After excluding records with missing or physiologically implausible split times, 78,912 participants were retained for analysis. Participants were included if they successfully completed the full marathon distance of 42.195 kilometers and had valid timing data recorded at all nine intermediate checkpoints. Records were excluded from analysis if any split time was missing, physiologically implausible (indicating timing system errors), or if the recorded finish time was inconsistent with checkpoint progression.

The demographic composition of the sample reflected the characteristics typical of major marathon events with qualifying standards. Participants ranged in age from 18 to 84 years, with a mean age of 42.4 years and a standard deviation of 11.4 years. The sample included 35,871 female runners (45.5%) and 43,041 male runners (54.5%), a sex distribution consistent with historical participation patterns at the Boston Marathon. Participants represented multiple countries, with the majority originating from the United States (81.0%), reflecting the domestic location of the event.

Finish times ranged from 2 hours 9 minutes to 8 hours 23 minutes, with a mean finish time of 3 hours 53 minutes and a median of 3 hours 46 minutes. The distribution of finish times was positively skewed, with the mode occurring in the 3:30–4:00 range. This distribution reflected the influence of qualifying standards, which effectively truncated the slow end of the performance spectrum compared to non-qualifying marathon events while still encompassing a wide range of performance levels from high-performance to recreational.

### Data collection and preprocessing

The dataset utilized in this study was obtained from the publicly available “Finishers Boston Marathon 2015, 2016 & 2017” dataset hosted on Kaggle (https://www.kaggle.com/datasets/rojour/boston-results). The temporal scope of 2015–2017 was therefore determined by the coverage of this curated public dataset rather than by arbitrary selection. This period was methodologically advantageous because it provided complete split-time data at all nine intermediate checkpoints, a large sample spanning three consecutive editions of the same race, and observations collected before the COVID-19 pandemic disrupted marathon participation patterns worldwide.

Race results were obtained from official Boston Athletic Association records, which are made publicly available following each annual event. For each participant, the following variables were extracted: bib number, age at race date, sex, country of residence, and cumulative elapsed time at each of nine intermediate timing points corresponding to the 5K, 10K, 15K, 20K, half-marathon (21.1K), 25K, 30K, 35K, and 40K distances, as well as the official finish time.

From the cumulative timing data, segment-specific split times were calculated for each of the nine race segments. Let $T_{i}$ denote the cumulative time recorded at checkpoint *i*, where i∈{1,2,…,9} corresponds to the 5K through 40K timing points, and let *T*_0_ = 0 represent the race start. The segment time $S_{i}$ for the *i*-th segment was computed as


$$\begin{array}{c} S_{i}=T_{i}-T_{i-1}, \end{array}$$ (1)

representing the time elapsed between consecutive checkpoints.

To enable comparison across runners of different ability levels, segment times were converted to segment-specific pace values expressed in minutes per kilometer. The pace $P_{i}$ for segment *i* was calculated by dividing the segment time by the segment distance $D_{i}$:


$$\begin{array}{c} P_{i}=\frac{S_{i}}{D_{i}}, \end{array}$$ (2)

where $D_{i}$ represents the distance in kilometers between checkpoints i−1 and *i*. Most segments spanned 5 kilometers, with the exception of the half-marathon checkpoint, which created segments of varying length that were appropriately accounted for in pace calculations.

Data quality procedures were implemented to identify and remove erroneous records. Segment paces exceeding 15 minutes per kilometer or falling below 2.5 minutes per kilometer were flagged as physiologically implausible and excluded from analysis. The upper threshold of 15 min/km (equivalent to 4 km/h, slower than typical walking speed) was selected because Boston Marathon participants have demonstrated qualification times well under this pace and because a sustained 15 min/km pace would be incompatible with the race’s approximate 6-hour cutoff. The lower threshold of 2.5 min/km exceeds current world-record marathon pace and likely reflects course-cutting or timing errors. Additionally, records with non-monotonic cumulative times (indicating a participant appeared to move backward on the course) were removed. After applying completeness and quality-control criteria, 726 records (0.9% of the original 79,638 records) were excluded, indicating high overall data quality in the retained analytic sample.

### Pacing strategy characterization

The characterization of pacing strategies employed both traditional ratio-based metrics and a data-driven pattern recognition approach to comprehensively capture the diversity of pacing behaviors in the sample.

Three complementary metrics were computed to quantify different aspects of pacing behavior for each participant. The first metric, termed the half-marathon pacing ratio and denoted *R*_half_, captured the overall distribution of effort between the first and second halves of the race. This ratio was defined as


$$\begin{array}{c} R_{\text{half}}=\frac{T_{\text{first half}}}{T_{\text{second half}}}, \end{array}$$ (3)

where *T*_first half_ represents the time elapsed from the start to the half-marathon checkpoint, and *T*_second half_ represents the time from the half-marathon to the finish. Values of *R*_half_ less than 1.0 indicate positive-splitting (faster first half), values equal to 1.0 indicate perfectly even pacing, and values greater than 1.0 indicate negative-splitting (faster second half). In the present study, however, *R*_half_ was used as a descriptive pacing metric, whereas the primary pattern classification was derived from cluster analysis of the full nine-dimensional normalized pacing profiles.

The second metric quantified the variability of pacing across race segments using the coefficient of variation of segment paces, denoted CV_pace_:


$$\begin{array}{c} {\text{CV}}_{\text{pace}}=\frac{\sigma_{P}}{\bar{P}}\times 100\%, \end{array}$$ (4)

where $\bar{P}$ represents the mean segment pace across all nine segments and $\sigma_{P}$ represents the standard deviation of segment paces. Higher values of CV_pace_ indicate greater pace variability throughout the race, while lower values indicate more consistent pacing.

The third metric characterized the trajectory of pace change throughout the race by computing the pace decay rate, denoted $\beta_{\text{pace}}$. This parameter was estimated by fitting a simple linear regression model to the sequence of segment paces as a function of segment number:


$$\begin{array}{c} P_{i}=\alpha +\beta_{\text{pace}}\cdot i+\varepsilon_{i}, \end{array}$$ (5)

where α represents the intercept (estimated initial pace), $\beta_{\text{pace}}$ represents the slope capturing the average change in pace per segment, *i* denotes the segment index, and $\varepsilon_{i}$ represents the residual error term. Positive values of $\beta_{\text{pace}}$ indicate progressive slowing (pace increasing, meaning slower running), while negative values indicate progressive acceleration.

To enable pattern recognition across runners with different overall ability levels, normalized pacing profiles were constructed for each participant. The normalized pace for segment *i*, denoted ${\tilde{P}}_{i}$, was computed as the ratio of the segment pace to the mean pace across all segments:


$$\begin{array}{c} {\tilde{P}}_{i}=\frac{P_{i}}{\bar{P}}, \end{array}$$ (6)

where $\bar{P}$ represents the arithmetic mean of segment paces for that individual. This normalization procedure removed the influence of overall running speed, allowing the analysis to focus on the shape of the pacing profile rather than absolute performance level. A normalized pace of 1.0 indicates running at exactly the mean pace, values below 1.0 indicate segments run faster than average, and values above 1.0 indicate segments run slower than average.

Each participant’s pacing profile was thus represented as a nine-dimensional vector $\tilde{𝐏}=({\tilde{P}}_{1},{\tilde{P}}_{2},\ldots ,{\tilde{P}}_{9})$, capturing the temporal evolution of relative pace throughout the race.

Distinct pacing patterns were identified using *K*-means cluster analysis applied to the normalized pacing profile vectors. The *K*-means algorithm partitions observations into *K* clusters by minimizing the within-cluster sum of squared Euclidean distances between observations and their assigned cluster centroids. Formally, the algorithm seeks to minimize the objective function


$$\begin{array}{c} J=\sum_{k=1}^{K}\sum_{\tilde{P}\in C_{k}}\|\tilde{P}-\mu_{k}{\|}^{2}, \end{array}$$ (7)

where $C_{k}$ represents the set of pacing profiles assigned to cluster *k*, $\mu_{k}$ represents the centroid (mean pacing profile) of cluster *k*, and ‖·‖ denotes the Euclidean norm.

The optimal number of clusters was determined through a combination of the elbow method and silhouette analysis. The elbow method involves plotting the within-cluster sum of squares as a function of the number of clusters and identifying the point at which additional clusters provide diminishing returns in variance reduction. The silhouette coefficient, ranging from −1 to +1, quantifies how similar each observation is to its own cluster compared to other clusters, with higher values indicating better-defined cluster structure. Based on these criteria, a four-cluster solution was selected as providing the optimal balance between cluster compactness and separation.

*K*-means clustering was selected over hierarchical clustering because the full dataset contained 78,912 observations, making a partitioning approach substantially more computationally efficient than quadratic-time hierarchical methods. In addition, our objective was to recover a prespecified four-pattern solution grounded in pacing theory rather than to construct a purely exploratory dendrogram. The cluster analysis procedure was implemented using the scikit-learn library in Python, with multiple random initializations (*n*_init_ = 10) employed to ensure convergence to a stable solution and reduce sensitivity to local minima. As a robustness check, Ward hierarchical clustering was also applied to a random 5,000-runner subsample during revision, yielding the same four qualitative pacing profiles with 72.7% label agreement relative to the *K*-means solution.

**Fig 1:**
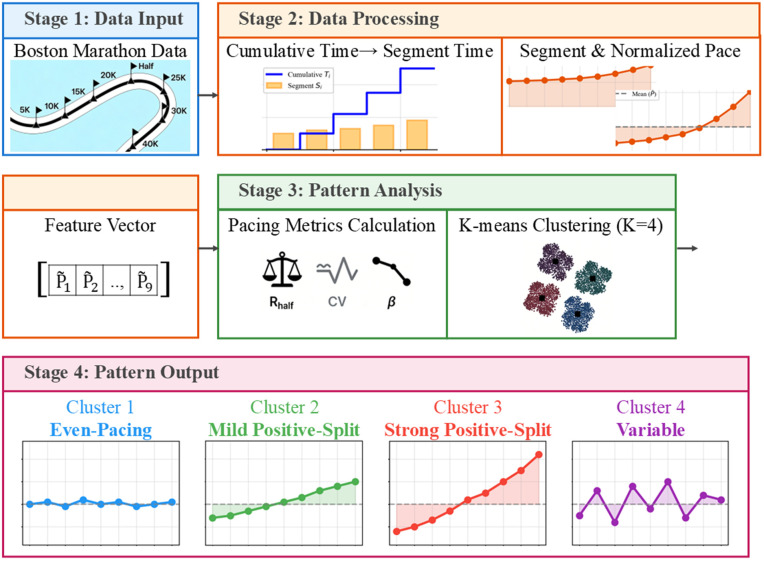
Analytical framework for pacing pattern identification. The workflow proceeds through four stages: (1) Data Input—Boston Marathon split time data from 79,638 finishers at nine checkpoints; (2) Processing—sequential transformation from cumulative times ($T_{i}$) to segment times ($S_{i}$), segment paces ($P_{i}$), and normalized pacing profiles (${\tilde{P}}_{i}$); (3) Analysis—computation of pacing metrics (half-ratio *R*, variability CV, decay rate β) and *K*-means clustering with *K* = 4; (4) Output—four distinct pacing patterns with characteristic pace trajectories: even-pacing (stable horizontal profile), mild positive-splitting (moderate late-race slowing), strong positive-splitting (pronounced late-race slowing), and variable-pacing (oscillating profile indicating inconsistent pace regulation).

The complete analytical workflow is presented in Fig 1, which illustrates the four-stage framework employed in this study. Stage 1 (Data Input) depicts the raw split time data from 79,638 Boston Marathon finishers across nine checkpoints. Stage 2 (Processing) shows the sequential transformation from cumulative times to segment times, segment paces, and finally normalized pacing profiles represented as nine-dimensional feature vectors. Stage 3 (Analysis) presents the dual analytical approach: pacing metrics calculation (half-marathon ratio *R*, coefficient of variation CV, and pace decay rate β) combined with *K*-means clustering to identify distinct patterns. Stage 4 (Output) displays the four identified pacing patterns—even-pacing, mild positive-splitting, strong positive-splitting, and variable-pacing—each illustrated with its characteristic normalized pace trajectory across race segments.

### Performance level stratification

To examine how pacing-performance relationships varied across the ability spectrum, participants were stratified into four performance level categories based on their official finish times. The stratification thresholds were selected to reflect meaningful distinctions in competitive level and physiological capacity, informed by Boston Marathon qualifying standards and established conventions in the marathon research literature.

The high-performance category encompassed runners with finish times under 3 hours, representing approximately the fastest 7% of the sample. While we acknowledge that true professional elite status typically requires faster times (under 2:15 for men and under 2:35 for women), the sub-3-hour threshold represents a widely recognized benchmark that distinguishes highly competitive amateur runners from recreational participants. In our sample, this threshold was achieved by 12.1% of male finishers and 0.9% of female finishers. The sub-elite category included runners finishing between 3:00 and 3:30, representing experienced and well-trained competitors who typically follow structured training programs. The recreational category comprised runners with finish times between 3:30 and 4:30, constituting the largest segment of the sample and representing serious amateur runners who train regularly but whose primary objectives may include personal achievement rather than competitive placement. The casual category included runners finishing in more than 4:30, representing participants whose goals may emphasize completion, charity fundraising, or social experience rather than time-based performance.

This stratification enabled separate analysis of pacing patterns and pacing-performance associations within each performance tier, addressing the possibility that optimal pacing strategies differ across ability levels due to differences in physiological capacity, racing experience, and event objectives.

### Statistical analysis

Statistical analyses were conducted to characterize pacing patterns, examine associations between pacing strategy and performance, and identify factors influencing pacing behavior. All analyses were performed using Python (version 3.9) with the NumPy, SciPy, and statsmodels libraries, with statistical significance evaluated at α=0.05.

Descriptive statistics were computed for all pacing metrics and demographic variables, with continuous variables summarized as means and standard deviations or medians and interquartile ranges as appropriate to their distributions, and categorical variables summarized as frequencies and percentages.

Comparisons of finish times across the four identified pacing pattern clusters were conducted using one-way analysis of variance (ANOVA), with the null hypothesis that mean finish times were equal across all clusters. When ANOVA indicated significant overall differences, post-hoc pairwise comparisons were performed using Tukey’s Honestly Significant Difference (HSD) test to identify which specific cluster pairs differed significantly while controlling the family-wise error rate. Effect sizes were quantified using eta-squared ($\eta^{2}$) for omnibus tests and Cohen’s *d* for pairwise comparisons, calculated as


$$\begin{array}{c} d=\frac{{\bar{X}}_{1}-{\bar{X}}_{2}}{s_{\text{pooled}}}, \end{array}$$ (8)

where ${\bar{X}}_{1}$ and ${\bar{X}}_{2}$ represent the means of the two groups being compared, and *s*_pooled_ represents the pooled standard deviation.

The distribution of pacing patterns across demographic groups (sex, age categories) and performance levels was examined using chi-square tests of independence. Standardized residuals were calculated to identify cells contributing disproportionately to significant chi-square statistics, indicating combinations of pacing pattern and demographic category that occurred more or less frequently than expected under the null hypothesis of independence.

Multiple linear regression analysis was employed to examine the relationship between pacing metrics and finish time while controlling for demographic covariates. The regression model took the form


$$\begin{array}{c} Y=\beta_{0}+\beta_{1}X_{\text{age}}+\beta_{2}X_{\text{sex}}+\beta_{3}X_{R_{\text{half}}}+\beta_{4}X_{\text{CV}}+\beta_{5}X_{\beta_{\text{pace}}}+\varepsilon , \end{array}$$ (9)

where *Y* represents finish time in minutes, *X*_age_ represents age in years, *X*_sex_ is a binary indicator for sex (coded as 0 for female and 1 for male), $X_{R_{\text{half}}}$ represents the half-marathon pacing ratio, *X*_CV_ represents the coefficient of variation of segment paces, $X_{\beta_{\text{pace}}}$ represents the pace decay rate, and ε represents the error term. The regression coefficients $\beta_{1}$ through $\beta_{5}$ quantify the association between each predictor and finish time after adjusting for the other variables in the model.

### “Hitting the wall” operationalization

The “hitting the wall” phenomenon was operationally defined as a substantial decline in running pace during the final portion of the race that exceeded normal fatigue-related slowing. Specifically, a participant was classified as having hit the wall if the pace in the final 7.195 kilometers (from 35K to finish) exceeded the mean pace for the first 35 kilometers by more than 20%. This threshold was selected based on previous research suggesting that pace declines exceeding this magnitude reflect qualitatively different physiological states associated with glycogen depletion rather than ordinary progressive fatigue.

Mathematically, let *P*_final_ represent the pace for the final race segment and ${\bar{P}}_{35}$ represent the mean pace across the first seven segments (through 35K). The “hitting the wall” indicator variable *H* was defined as


$$\begin{array}{c} H=\left\{ \begin{array}{cc} 1 & \text{if }\frac{P_{\text{final}}-{\bar{P}}_{35}}{{\bar{P}}_{35}}>0.20,\\ 0 & \text{otherwise.} \end{array}\right. \end{array}$$ (10)

The incidence of hitting the wall was calculated overall and stratified by pacing pattern cluster, with chi-square tests used to evaluate whether the proportion of runners hitting the wall differed significantly across pacing strategies. Logistic regression was employed to estimate the odds ratio for hitting the wall associated with each pacing pattern relative to even-pacing as the reference category, adjusting for age, sex, and overall finish time.

## Results

### Participant characteristics

The final analytical sample comprised 78,912 marathon finishers with complete split time data across three consecutive Boston Marathon events (2015–2017). Table 1 presents the demographic characteristics and performance distributions of the study sample, stratified by sex.

**Table 1:** Demographic characteristics and performance distributions of study participants (N = 78,912).

Characteristic	Male (n = 43,041)	Female (n = 35,871)	Total (N = 78,912)
*Age (years)*			
Mean ± SD	44.6 ± 11.5	39.8 ± 10.6	42.4 ± 11.4
Range	18–83	18–84	18–84
*Finish time (hh:mm:ss)*			
Mean ± SD	3:43:24 ± 0:41:39	4:04:15 ± 0:37:50	3:52:53 ± 0:41:17
Median (IQR)	3:35:00 (3:13–4:05)	3:55:32 (3:37–4:23)	3:46:19 (3:24–4:15)
Range	2:09:17–8:23:27	2:21:52–7:59:33	2:09:17–8:23:27
*Performance level, n (%)*			
High-performance (<3:00)	5,208 (12.1%)	325 (0.9%)	5,533 (7.0%)
Sub-elite (3:00–3:30)	14,084 (32.7%)	5,208 (14.5%)	19,292 (24.4%)
Recreational (3:30–4:30)	17,778 (41.3%)	22,763 (63.5%)	40,541 (51.4%)
Casual (>4:30)	5,971 (13.9%)	7,575 (21.1%)	13,546 (17.2%)
*Country of origin, n (%)*			
United States	34,847 (81.0%)	29,041 (81.0%)	63,888 (81.0%)
International	8,194 (19.0%)	6,830 (19.0%)	15,024 (19.0%)

SD = standard deviation; IQR = interquartile range.

The sample exhibited the expected sex differences in marathon performance, with male runners demonstrating significantly faster mean finish times than female runners (3:43:24 vs. 4:04:15, *t* = 104.5, *p* < 0.001, Cohen’s *d* = 0.52). The age distribution showed males were slightly older on average (44.6 vs. 39.8 years). The majority of participants (51.4%) fell within the recreational performance category, with smaller proportions classified as high-performance (7.0%), sub-elite (24.4%), or casual (17.2%). Female runners were proportionally overrepresented in the casual and recreational categories and underrepresented in the high-performance and sub-elite categories, reflecting established sex differences in marathon performance distributions.

### Pacing pattern identification

Application of *K*-means cluster analysis to the normalized pacing profiles yielded four distinct clusters that corresponded closely to the theoretically anticipated pacing strategy categories. Table 2 summarizes the pacing metrics and performance characteristics of each identified cluster.

**Table 2:** Pacing metrics and performance characteristics of identified pacing pattern clusters.

Metric	Even-Pacing	Mild Positive-Split	Strong Positive-Split	Variable-Pacing
	(n = 37,827)	(n = 25,135)	(n = 9,600)	(n = 6,350)
Proportion of sample	47.9%	31.9%	12.2%	8.0%
*Pacing metrics (Mean ± SD)*				
Half-ratio *R*_half_	0.95 ± 0.03	0.87 ± 0.03	0.82 ± 0.05	0.73 ± 0.06
CV_pace_ (%)	3.7 ± 1.8	8.7 ± 2.3	12.3 ± 3.7	19.8 ± 6.1
Pace decay β (s/km/seg)	2.7 ± 2.3	9.1 ± 2.8	14.9 ± 5.2	22.4 ± 7.7
*Performance (Mean ± SD)*				
Finish time (min)	216.9 ± 33.5	234.3 ± 35.8	276.4 ± 44.1	256.6 ± 39.3
First half (min)	105.9	108.8	124.7	108.1
Second half (min)	111.0	125.5	151.7	148.5
*Demographics*				
Male (%)	50.7%	57.5%	46.1%	78.2%
Mean age (years)	41.8 ± 10.9	42.9 ± 11.4	43.2 ± 12.3	43.0 ± 12.1

CV = coefficient of variation; β = pace decay rate (positive values indicate slowing).

The even-pacing cluster was the largest identified pattern, comprising 47.9% of participants (*n* = 37,827). This cluster was characterized by relatively high half-marathon ratios ($R_{\text{half}}=0.95\pm 0.03$), minimal pace variability (${\text{CV}}_{\text{pace}}=3.7\%\pm 1.8\%$), and low pace decay (β=2.7±2.3 s/km/segment). Runners in this cluster maintained remarkably consistent pacing throughout the race, with normalized segment paces deviating less than 5% from the individual mean across all nine checkpoints.

The mild positive-split cluster encompassed 31.9% of participants (*n* = 25,135). This cluster exhibited faster first halves followed by relatively controlled late-race slowing: half-marathon ratios of $R_{\text{half}}=0.87\pm 0.03$, moderate pace variability (${\text{CV}}_{\text{pace}}=8.7\%\pm 2.3\%$), and notable pace decay (β=9.1±2.8 s/km/segment). The mean second-half time exceeded the first-half time by 16.7 minutes in this cluster.

The strong positive-split cluster comprised 12.2% of participants (*n* = 9,600) and demonstrated aggressive early pacing followed by substantial late-race slowing. This cluster showed low half-marathon ratios ($R_{\text{half}}=0.82\pm 0.05$), high pace variability (${\text{CV}}_{\text{pace}}=12.3\%\pm 3.7\%$), and pronounced pace decay rates (β=14.9±5.2 s/km/segment). The mean second-half time exceeded the first-half time by 27.0 minutes.

The variable-pacing cluster included 8.0% of participants (*n* = 6,350) and was distinguished by extremely high pace variability (${\text{CV}}_{\text{pace}}=19.8\%\pm 6.1\%$) and the most severe pace decay (β=22.4±7.7 s/km/segment). The half-marathon ratio in this cluster was very low ($R_{\text{half}}=0.73\pm 0.06$), indicating aggressive early pacing combined with substantial within-race fluctuations. Notably, this cluster had the highest proportion of male runners (78.2%).

**Fig 2:**
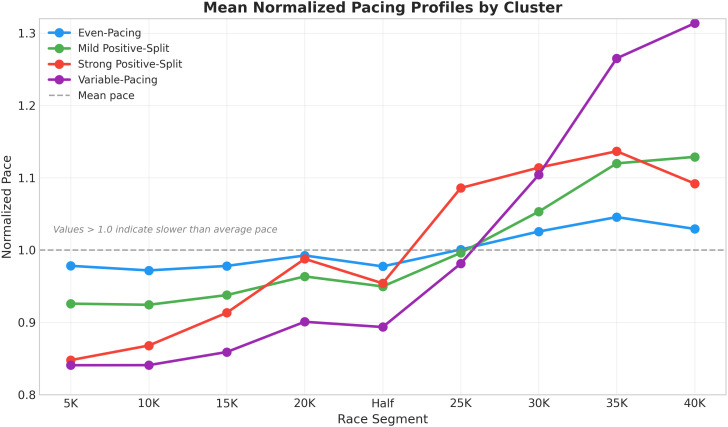
Mean normalized pacing profiles for the four identified pacing pattern clusters. Each line represents the cluster centroid, with normalized pace values (y-axis) plotted against race segment (x-axis). The horizontal dashed line at 1.0 represents the individual mean pace. Even-pacing (blue) maintains values close to 1.0 throughout; mild positive-splitting (green) shows moderate progressive increase indicating controlled slowing; strong positive-splitting (red) shows steeper progressive increase indicating pronounced slowing; variable-pacing (purple) exhibits irregular fluctuations.

Fig 2 displays the mean normalized pacing profiles for each cluster, illustrating the distinctive shape characteristics that differentiate the four pacing strategies. The profiles clearly demonstrate the stable trajectory of even-pacing, the moderate elevation (slowing) characteristic of mild positive-splitting, the steeper elevation characteristic of strong positive-splitting, and the oscillatory pattern of variable-pacing.

To facilitate comparison with prior literature that employed predefined half-marathon ratio thresholds, we also classified participants using conventional criteria based on *R*_half_ alone: even-pacing ($0.95\leq R_{\text{half}}\leq 1.05$), positive-splitting (*R*_half_ < 0.95), and negative-splitting (*R*_half_ > 1.05). This threshold-based approach classified 24.0% of runners as even-pacing, 75.6% as positive-splitting, and only 0.4% as negative-splitting. Agreement between the traditional three-category taxonomy and a collapsed version of our cluster-derived solution was moderate (Cohen’s κ=0.511; overall agreement = 76.0%). The main discrepancy was that the variable-pacing cluster was uniformly classified as positive-splitting by *R*_half_ alone, highlighting the added value of segment-level variability information in distinguishing qualitatively different pacing profiles.

### Pacing patterns and performance

The relationship between pacing pattern and marathon performance was examined through comparison of finish times across the four identified clusters. One-way ANOVA revealed highly significant differences in mean finish time among pacing patterns (*F*_3,78908_ = 8012.1, *p* < 0.001, $\eta^{2}=0.233$). Table 3 presents the pairwise comparisons with effect sizes.

**Table 3:** Pairwise comparisons of finish times between pacing pattern clusters.

Comparison	Mean Diff (min)	95% CI	Cohen’s *d*	*p*-value
Even vs. Mild Positive-Split	−17.4	[-17.9, -16.8]	0.50	<0.001
Even vs. Strong Positive-Split	−59.5	[-60.3, -58.6]	1.65	<0.001
Even vs. Variable-Pacing	−39.7	[-40.6, -38.8]	1.15	<0.001
Mild Positive-Split vs. Strong Positive-Split	−42.1	[-43.0, -41.2]	1.10	<0.001
Mild Positive-Split vs. Variable-Pacing	−22.3	[-23.3, -21.3]	0.61	<0.001
Strong Positive-Split vs. Variable-Pacing	19.8	[18.4, 21.1]	0.47	<0.001

Mean Diff = difference in mean finish time (negative values indicate faster times for the first-listed pattern); CI = confidence interval. All *p*-values Bonferroni-corrected.

Even-pacing was associated with the fastest mean finish time (216.9 ± 33.5 minutes), significantly outperforming all other pacing strategies. Compared with the strong positive-split cluster, even-pacing runners finished 59.5 minutes faster on average (Cohen’s *d* = 1.65, a large effect size). The performance advantage of even-pacing over variable-pacing was 39.7 minutes (*d* = 1.15). The mild positive-split cluster was the second-fastest pattern, with finish times 17.4 minutes slower than even-pacing but 42.1 minutes faster than the strong positive-split cluster. Strong positive-splitting runners finished 19.8 minutes slower than variable-pacing runners (*d* = 0.47), indicating that variable-pacing and strong positive-splitting were the patterns most consistently associated with impaired performance.

**Fig 3:**
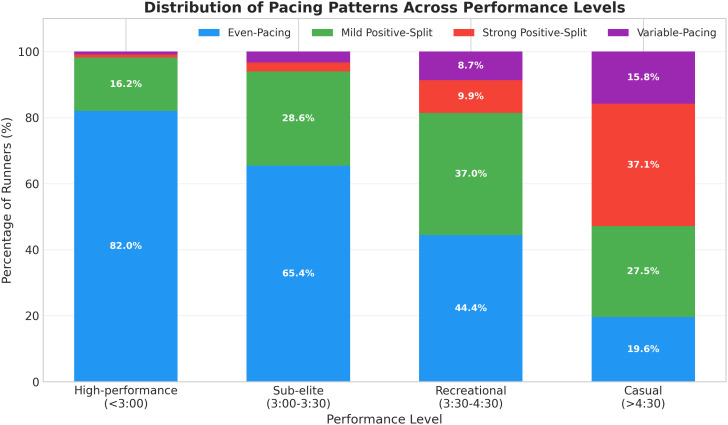
Distribution of pacing patterns across performance levels. The stacked bar chart shows the proportion of runners adopting each pacing strategy within high-performance (<3:00), sub-elite (3:00–3:30), recreational (3:30–4:30), and casual (>4:30) performance categories. Even-pacing prevalence decreases progressively from high-performance to casual runners, while strong positive-splitting becomes more common.

The distribution of pacing patterns varied substantially across performance levels, as illustrated in Fig 3. Chi-square analysis confirmed significant differences in pacing pattern prevalence across performance categories ($\chi^{2}=16,876.4$, *df* = 9, *p* < 0.001, Cramér’s *V* = 0.27).

High-performance runners (<3:00 finish time) demonstrated the highest prevalence of even-pacing (82.0%) and the lowest prevalence of strong positive-splitting (1.0%). In contrast, casual runners (>4:30) showed markedly different patterns, with only 19.6% adopting even-pacing while 37.1% exhibited strong positive-splitting. This gradient was monotonic across performance levels: even-pacing prevalence decreased from 82.0% (high-performance) to 65.4% (sub-elite) to 44.4% (recreational) to 19.6% (casual), while strong positive-splitting increased correspondingly from 1.0% to 2.7% to 9.9% to 37.1%.

The performance advantage of even-pacing was consistent across all performance levels but varied in magnitude. Among high-performance runners, even-pacing was associated with finish times 4.6 minutes faster than strong positive-splitting (*d* = 0.53). This advantage was 3.6 minutes among sub-elite (*d* = 0.41), 13.2 minutes among recreational (*d* = 0.86), and 13.6 minutes among casual runners (*d* = 0.51). The larger effect sizes in recreational categories suggest that pacing errors are particularly consequential for mid-level runners who may have less experience calibrating their pace.

#### Sex-specific quartile sensitivity analysis.

To address the concern that fixed time thresholds may not fully account for sex differences in performance, we repeated the performance-level analysis using sex-specific quartiles rather than absolute finish-time cutoffs. Within each sex, runners were classified into four groups based on within-sex finish-time quartiles (Q1: fastest 25%, Q2: 25–50%, Q3: 50–75%, Q4: slowest 25%). The association between even-pacing and superior performance remained robust across both sexes: even-pacing prevalence declined monotonically from Q1 to Q4 for male runners (75.0%, 50.2%, 35.2%, and 17.9%, respectively) and for female runners (81.3%, 60.2%, 41.1%, and 25.2%, respectively). Chi-square tests confirmed strong associations between performance quartile and pacing pattern for both male runners ($\chi^{2}=11,269.0$, *p* < 0.001) and female runners ($\chi^{2}=9,792.2$, *p* < 0.001), indicating that the main findings were not artifacts of the fixed threshold approach.

Sex differences in pacing pattern adoption were statistically significant ($\chi^{2}=2,023.0$, *df* = 3, *p* < 0.001) with moderate magnitude (Cramér’s *V* = 0.16). Male runners exhibited higher rates of variable-pacing (11.5% vs. 3.9%) and lower rates of even-pacing (44.6% vs. 52.0%) compared to female runners. Interestingly, female runners showed higher rates of strong positive-splitting (14.4% vs. 10.3%), possibly reflecting different race strategies or experience levels. These findings suggest complex sex-based differences in pacing behavior.

Age was significantly associated with pacing pattern adoption ($\chi^{2}=362.2$, *p* < 0.001). Among runners in the 35–50 age group, even-pacing prevalence was highest (50.1%) compared to 49.1% among runners under 35 and 42.7% among runners over 50. Correspondingly, strong positive-splitting prevalence was lowest in the 35–50 group (10.9%) compared to 12.3% in younger runners and 14.2% in older runners. These patterns suggest that mid-career runners may have optimal pacing discipline, while both younger and older runners show somewhat higher rates of suboptimal pacing strategies.

### Predictors of pacing strategy and performance

Multiple linear regression analysis examined the joint effects of pacing metrics and demographic variables on finish time. Table 4 presents the regression coefficients and model statistics.

**Table 4:** Multiple linear regression predicting marathon finish time from pacing metrics and demographics.

Predictor	β	SE	*t*	*p*
Intercept	−429.91	4.06	−105.8	<0.001
Age (years)	0.69	0.01	80.5	<0.001
Sex (male = 1)	−20.67	0.20	−104.5	<0.001
Half-ratio *R*_half_	635.58	4.12	154.3	<0.001
CV_pace_ (%)	−0.78	0.05	−16.3	<0.001
Pace decay β (s/km/seg)	10.63	0.05	218.8	<0.001
Model statistics: *R*^2^ = 0.603, Adjusted *R*^2^ = 0.603, *F*_5,78906_ = 23,987, *p* < 0.001				

β = unstandardized regression coefficient; SE = standard error. Dependent variable is finish time in minutes.

The regression model explained 60.3% of variance in finish time (*R*^2^ = 0.603, *F*_5,78906_ = 23,987, *p* < 0.001). All predictors were statistically significant. The half-marathon ratio showed a strong positive association with finish time: each 0.01-unit increase in *R*_half_ (indicating less positive-splitting) was associated with 6.36 minutes faster finish time, controlling for other variables. The pace decay rate was strongly associated with finish time, with each 1 s/km/segment increase in $\beta_{\text{pace}}$ associated with 10.63 minutes slower finish time, reflecting the performance cost of late-race slowing.

Demographic predictors showed expected patterns: male sex was associated with 20.67 minutes faster finish times, while each additional year of age was associated with 0.69 minutes slower performance. The pacing metrics remained significantly associated with finish time after controlling for these demographic factors, indicating that pacing behavior captured meaningful variation in performance beyond these measured covariates, while not establishing an independent causal effect.

As a robustness check, we re-estimated the regression model with year included as a fixed-effect covariate (2015 as the reference year). The year coefficients were small in magnitude ($\beta_{2016}=0.41$ min, *p* = 0.080; $\beta_{2017}=1.68$ min, *p* < 0.001), and the model fit changed negligibly (*R*^2^ from 0.6032 to 0.6035; $\Delta R^{2}=0.0003$). Importantly, the coefficients for the pacing metrics and demographic covariates remained virtually unchanged, indicating that the main findings were robust to modest year-to-year variation.

### “Hitting the wall” analysis

Application of the operational definition for “hitting the wall” (final segment pace exceeding the mean pace for the first 35K by more than 20%) classified 11,973 runners (15.2% of the sample) as having experienced this phenomenon. The incidence of hitting the wall varied dramatically across pacing pattern clusters, as shown in Table 5.

**Table 5:** Incidence of “hitting the wall” by pacing pattern cluster.

Pacing Pattern	n	Hit Wall (%)	OR (95% CI)	*p*-value
Even-Pacing	37,827	65 (0.2%)	1.00 (ref)	—
Mild Positive-Split	25,135	5,365 (21.3%)	99.6 (77.9–127.3)	<0.001
Strong Positive-Split	9,600	1,122 (11.7%)	48.6 (37.8–62.4)	<0.001
Variable-Pacing	6,350	5,421 (85.4%)	2,129.7 (1,653.6–2,743.0)	<0.001
Total	78,912	11,973 (15.2%)	—	—

OR = odds ratio from logistic regression adjusted for age, sex, and finish time; CI = confidence interval; ref = reference category.

Even-pacing runners demonstrated remarkably low incidence of hitting the wall at only 0.2%, while variable-pacing runners experienced the highest incidence at 85.4%. The odds of hitting the wall were dramatically higher for all other patterns compared to even-pacing. Variable-pacing showed extremely elevated risk (OR = 2,129.7, 95% CI: 1,653.6–2,743.0, *p* < 0.001), reflecting the severe late-race pace decline characteristic of this pattern. Mild positive-splitting (OR = 99.6, 95% CI: 77.9–127.3) and strong positive-splitting (OR = 48.6, 95% CI: 37.8–62.4) also showed substantially elevated odds.

**Fig 4:**
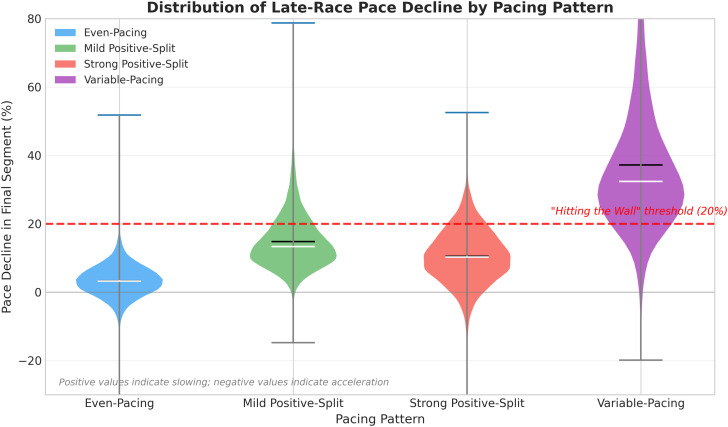
Distribution of final segment pace decline across pacing patterns. Violin plots show the distribution of percentage pace change from mean first-35K pace to final segment pace for each pacing pattern. The horizontal dashed line at 20% represents the “hitting the wall” threshold. Positive values indicate slowing; negative values indicate acceleration. Even-pacing shows a narrow distribution centered near zero, while variable-pacing exhibits a wide, right-skewed distribution with substantial mass above the 20% threshold.

Fig 4 illustrates the distribution of pace decline in the final segment across pacing patterns, demonstrating the stark contrast between even-pacing and variable-pacing strategies.

The relationship between early pacing aggressiveness and hitting the wall risk was further examined by analyzing the pace at the 10K checkpoint relative to average pace. Runners whose early pace (first two segments) was more than 5% faster than their overall average pace had a 26.6% incidence of hitting the wall, compared to only 1.5% among runners whose early pace was within the average range. This dose-response relationship underscores the critical importance of restraint in the early race stages for avoiding late-race collapse.

Among runners who hit the wall, the mean pace decline in the final segment was 33.1% compared to the first 35K average. This corresponded to a mean slowdown of 1:48 per kilometer in the final segment, underscoring the practical performance relevance of severe late-race fatigue. A sensitivity analysis using alternative thresholds of 15% and 25% pace decline yielded consistent conclusions: overall incidence was 24.5% and 9.6%, respectively, even-pacing remained near zero (1.2% and 0.0%), and variable-pacing remained markedly elevated (93.4% and 72.8%). The between-pattern differences remained highly significant at each threshold ($\chi^{2}=31,195.9$ to 33,677.1, all *p* < 0.001), indicating that the main interpretation was robust to reasonable variation in the operational definition.

## Discussion

This large-scale analysis of pacing strategies among 78,912 Boston Marathon finishers yielded several important findings that advance our understanding of pace distribution in marathon running. The application of data-driven cluster analysis to segment-by-segment split times successfully identified four distinct pacing patterns: even-pacing, mild positive-splitting, strong positive-splitting, and variable-pacing. These findings confirm our first hypothesis (H1) and validate the utility of unsupervised learning methods for identifying naturally occurring pacing behaviors in large athletic populations.

The results demonstrated a clear and consistent association between pacing strategy and marathon performance, with even-pacing emerging as the pattern most strongly associated with favorable finish times across demographic subgroups and performance levels. Runners adopting even-pacing strategies finished an average of 59.5 minutes faster than those in the strong positive-split cluster, with effect sizes in the large range (Cohen’s *d* = 1.65). This performance advantage was observed among both high-performance and recreational runners, though the patterns varied by performance level. These findings strongly support our second hypothesis (H2) regarding the performance advantages associated with even-pacing.

The prevalence of pacing patterns varied systematically with performance level, with high-performance runners demonstrating substantially higher rates of even-pacing adoption (82.0%) compared to casual participants (19.6%). Conversely, strong positive-splitting was more prevalent among casual runners (37.1%) compared to high-performance runners (1.0%). This gradient confirms our third hypothesis (H3) and suggests that superior pacing discipline may be both a marker and a correlate of higher performance status.

Perhaps most consequentially, the analysis revealed a dramatic relationship between pacing strategy and the incidence of “hitting the wall.” Runners adopting variable-pacing strategies experienced this severe late-race phenomenon at remarkably high rates (85.4%), while even-pacing runners showed near-zero incidence (0.2%), confirming our fourth hypothesis (H4). The sensitivity analysis across 15%, 20%, and 25% thresholds showed the same rank ordering of patterns, underscoring that this conclusion is not an artifact of the specific cutoff used in the primary analysis.

The present findings are broadly consistent with prior research on marathon pacing while extending knowledge in several important respects. The identification of even-pacing as the pattern most consistently associated with favorable outcomes aligns with the theoretical framework established by Abbiss and Laursen [1] and empirical observations from elite marathon performance [3,16]. However, the current study provides large-scale evidence that this association holds across the full spectrum of marathon participants, from high-performance competitors to casual finishers.

The prevalence of strong positive-splitting observed in this study (12.2% of all participants) was lower than some previous reports, likely reflecting the qualifying nature of the Boston Marathon sample, which selects for more experienced runners [12,18]. Nevertheless, when combined with variable-pacing (8.0%), approximately 20% of runners exhibited pacing patterns characterized by aggressive starts or marked within-race instability. The sex differences in pacing pattern adoption showed a nuanced pattern, with male runners more prone to variable-pacing (11.5% vs. 3.9%) while female runners showed higher strong positive-splitting rates (14.4% vs. 10.3%), suggesting complex behavioral factors in pacing decisions.

The “hitting the wall” incidence of 15.2% observed in this study is substantially lower than the 40% estimate reported by Buman and colleagues [6], likely reflecting differences in operational definitions and the qualifying nature of the Boston Marathon sample. Nevertheless, the strong association between pacing strategy and hitting-the-wall risk confirms and extends the metabolic modeling predictions of Rapoport [8], demonstrating in a large empirical sample that conservative early pacing is consistently associated with lower rates of severe late-race slowing.

The gradient of even-pacing prevalence across performance levels—from 82.0% among high-performance runners to 19.6% among casual participants—represents a strong finding that underscores the relationship between pacing discipline and performance. This pattern suggests that pacing skill develops with experience and competitive level, though the cross-sectional design of this study precludes determination of whether improved pacing leads to better performance or whether faster runners simply have more refined pacing abilities.

The performance advantages of even-pacing and the risks of positive-splitting can be understood through established principles of exercise physiology. Marathon running is fundamentally constrained by the finite availability of glycogen stores in skeletal muscle and the liver, which provide the primary fuel source for sustained high-intensity exercise [8,32]. Running at intensities above the sustainable threshold accelerates glycogen depletion disproportionately, creating a metabolic debt that manifests as severe fatigue in the later stages of the race [8].

Positive-splitting strategies, characterized by fast early pacing followed by progressive slowing, reflect a mismatch between initial running intensity and physiological capacity. Runners who start too fast relative to their sustainable pace accumulate metabolic byproducts—including lactate, hydrogen ions, and inorganic phosphate—that impair muscular function and contribute to peripheral fatigue [23]. Additionally, the elevated carbohydrate oxidation rates associated with aggressive early pacing deplete glycogen stores more rapidly, precipitating the metabolic crisis characteristic of “hitting the wall” [7,8].

The central governor model proposed by Noakes and colleagues [14,23] provides a complementary explanation for the observed pacing patterns. According to this framework, the brain continuously monitors peripheral physiological status and adjusts exercise intensity to prevent catastrophic homeostatic failure. Experienced runners may have better-calibrated central governors that enable more accurate pacing from the race onset, while less experienced participants may override these protective signals in the excitement of the early race stages, only to pay a severe performance penalty later [18,25].

The age-related differences in pacing pattern adoption—with older runners demonstrating more conservative strategies—may reflect both accumulated racing experience and age-related changes in the central regulation of exercise intensity [12,25,27]. Older athletes may have learned through repeated marathon participation that restraint in early stages yields superior overall performance, or they may be more responsive to the conservative signals from the central governor system [25,27].

The findings of this study have practical implications for marathon runners, coaches, and race organizers seeking to improve pacing education and participant experience. For runners at all levels, the results suggest that restraint in the early race stages is associated with faster finish times and a lower incidence of “hitting the wall.” The quantitative relationships documented here can inform the development of individualized pacing plans, while recognizing that the current design does not permit causal inference.

For coaches and training professionals, the systematic differences in pacing behavior across performance levels suggest that pacing discipline should be explicitly addressed in marathon preparation. Novice marathoners, who are most prone to strong positive-splitting, may particularly benefit from structured pacing practice during long training runs and tune-up races. The use of GPS watches and real-time pace feedback can help runners develop the kinesthetic awareness necessary for maintaining consistent effort throughout the race.

Race organizers may consider the findings in designing participant support structures. The observation that “hitting the wall” is concentrated among positive-splitting-derived patterns suggests that medical and aid station resources should be positioned to address the predictable surge in distressed participants during the final 10 kilometers. Educational materials distributed to participants before the race could emphasize the importance of conservative early pacing, potentially reducing the incidence of severe late-race difficulties.

The sex differences in pacing pattern adoption, with male runners more prone to aggressive early pacing, suggest that targeted messaging about pacing restraint may be particularly valuable for male participants. Similarly, the lower rates of even-pacing among younger runners indicate that less experienced marathoners would benefit from explicit guidance about the importance of measured early pacing.

Several limitations of this study warrant consideration when interpreting the findings. First, the cross-sectional observational design—in which each participant was measured at a single race event rather than followed across multiple races—precludes causal inference about the relationship between pacing strategy and performance. Although even-pacing was consistently associated with faster finish times in our analyses, we cannot determine whether this pattern reflects a direct benefit of pacing itself, the influence of underlying factors such as fitness, training quality, and racing experience, or a bidirectional relationship in which better-prepared runners pace more evenly and also perform better overall. Accordingly, pacing strategy in this context should be interpreted not only as a potentially meaningful behavioral pattern, but also as a marker of underlying ability that was not fully captured in the available data. We note that while the overall design is cross-sectional, the segment-by-segment split time data have a within-race temporal structure that enables analysis of pace progression patterns within each race performance. Second, the Boston Marathon sample, while large and demographically diverse, may not generalize to all marathon populations. The qualifying requirements of the Boston Marathon ensure that participants have demonstrated a certain level of endurance running competence, potentially excluding the slowest and least experienced runners who might exhibit different pacing behaviors. Additionally, the point-to-point course with a net downhill profile, including the demanding “Heartbreak Hill” section, may influence pacing behaviors in ways that differ from flat or loop courses. Future research should examine pacing patterns in non-qualifying marathon events with different course profiles to assess generalizability. Third, the study lacked information about factors that might influence pacing strategy selection, including training history, previous marathon experience, race-day weather conditions, and course topography awareness. Fourth, the operational definition of “hitting the wall” based on pace decline thresholds, while consistent with previous research, may not perfectly capture the subjective experience of glycogen depletion. Some runners may experience substantial perceived fatigue without meeting the 20% pace decline threshold, while others may slow for tactical or mechanical reasons unrelated to metabolic crisis. Finally, although inclusion of year fixed effects changed the regression results only minimally, aggregation across 2015–2017 may still obscure subtle temporal trends in pacing behavior. Longitudinal or intervention-based studies would be needed to determine whether modifying pacing behavior can improve performance independent of baseline fitness and experience.

## Conclusion

This large-scale analysis of marathon pacing strategies provides robust evidence that even-pacing was the pacing pattern most consistently associated with favorable performance outcomes across the full spectrum of marathon participants. Among 78,912 Boston Marathon finishers, runners maintaining consistent pace throughout the race finished an average of 59.5 minutes faster than those in the strong positive-split cluster. The association between pacing strategy and performance was consistent across sex, age, and performance level categories, with the regression model explaining 60.3% of variance in finish time.

The study identified four distinct pacing patterns through data-driven cluster analysis: even-pacing, mild positive-splitting, strong positive-splitting, and variable-pacing. Even-pacing emerged as the dominant pattern (47.9%), particularly among high-performance runners (82.0%). Variable-pacing, while affecting only 8.0% of the sample, was associated with dramatically elevated risk of “hitting the wall” (85.4%), compared to near-zero incidence (0.2%) among even-pacing runners.

These findings have important practical implications for marathon preparation. Runners at all levels may benefit from prioritizing pacing discipline in race planning, with particular emphasis on restraint during the early race stages when the temptation to run faster than sustainable pace is greatest. Coaches may likewise consider explicit pacing training, especially for novice runners who are most vulnerable to pacing errors. The quantitative relationships documented here can inform the development of evidence-based pacing guidelines tailored to individual characteristics and performance objectives.

Future research should employ longitudinal designs to examine whether pacing skill improves with marathon experience and whether targeted pacing interventions can reduce the incidence of strong positive-splitting and “hitting the wall” among recreational participants. Investigation of the psychological factors that contribute to aggressive early pacing—including competitive arousal, goal orientation, and risk tolerance—may yield additional insights for developing effective pacing interventions. The integration of real-time physiological monitoring with pacing feedback represents a promising avenue for technology-assisted pacing optimization in marathon running.
